# Microglial aging in the healthy CNS: phenotypes, drivers, and rejuvenation

**DOI:** 10.3389/fncel.2013.00022

**Published:** 2013-03-13

**Authors:** Wai T. Wong

**Affiliations:** Unit on Neuron-Glia Interactions in Retinal Disease, National Eye Institute, National Institutes of HealthBethesda, MD, USA

**Keywords:** microglia, aging, priming, neurodegeneration, rejuvenation, inflammation, activation, senescence

## Abstract

Neurodegenerative diseases such as Alzheimer's disease, Parkinson's disease, and age-related macular degeneration (AMD), share two characteristics in common: (1) a disease prevalence that increases markedly with advancing age, and (2) neuroinflammatory changes in which microglia, the primary resident immune cell of the CNS, feature prominently. These characteristics have led to the hypothesis that pathogenic mechanisms underlying age-related neurodegenerative disease involve aging changes in microglia. If correct, targeting features of microglial senescence may constitute a feasible therapeutic strategy. This review explores this hypothesis and its implications by considering the current knowledge on how microglia undergo change during aging and how the emergence of these aging phenotypes relate to significant alterations in microglial function. Evidence and theories on cellular mechanisms implicated in driving senescence in microglia are reviewed, as are “rejuvenative” measures and strategies that aim to reverse or ameliorate the aging microglial phenotype. Understanding and controlling microglial aging may represent an opportunity for elucidating disease mechanisms and for formulating novel therapies.

## Introduction

As the predominant resident immune cell in the central nervous system (CNS), microglia has been causally linked to the development of neurodegenerative diseases in which neuroinflammation plays a prominent role. As the prevalence of such diseases, which include Alzheimer's disease, Parkinson's disease, and age-related macular degeneration (AMD), demonstrates a strong association with increasing age, aging changes in microglia have been hypothesized to play a prominent role in disease pathogenesis. Over the last few years, new and exciting discoveries with respect to microglial physiology and function have provided key insights that provide a conceptual framework for thinking about the connection between microglial aging and neurodegenerative disease. First, we have learned from studies employing parabiosis to create chimerism in circulating bone-marrow derived precursors that microglia are long-lived cells in the healthy CNS, with residence times that extend across much of an animal's life-span, and are therefore susceptible to aging factors *in situ* (Ajami et al., 2007; Mildner et al., 2007). Second, a critical mass of studies focused on characterizing microglia in the aged CNS have elucidated a combination of anatomical, morphological, physiological, and molecular changes exhibited by aging microglia that now provides a comprehensive depiction of the senescent microglial phenotype. Third, a combination of detailed ultrastructure studies and live-cell imaging techniques has provided novel insights into the constitutive cellular interactions conducted by microglia through which the everyday functional roles of microglia can be discerned. These data allow us to conceptually connect the features of the aging microglial phenotype with significant and consequential alterations in microglial function in a way that is helpful for thinking about mechanisms of disease pathogenesis and for formulating strategies of disease prevention and treatment.

This review aims to summarize what is currently known about: (1) the nature of the aging microglial phenotype, (2) how microglial aging can impact the function of microglia in their everyday roles, as well as in their response to perturbations, (3) what mechanisms underlie the generation of the aging microglial phenotype, and (4) whether aging changes in microglia may be successfully reversed (i.e., if aged microglia may be therapeutically rejuvenated).

## The aging phenotype of microglia

### Aging changes in microglial density, distribution, and ramified morphology

How do microglia in the uninjured and healthy CNS change with aging? Microglia in the healthy young CNS have a typical ramified morphology and are distributed throughout the neural parenchyma in a “space-filling” manner, providing efficient spatial coverage of the entire CNS milieu. This orderly arrangement of ramified microglia reflects the presence of organizing factors whose identity, mechanism, and purpose are still rather mysterious. Interestingly, as the CNS ages, the fidelity of these regulatory factors appears to diminish as the numbers, distribution, and morphological features of microglia progressively changes with time, reflecting aging-sensitive alterations in microglia function.

The total number and density of microglia have been noted to increase significantly with age in various CNS compartments, including the hippocampus (Mouton et al., 2002), visual and auditory cortices (Tremblay et al., 2012), and the retina (Damani et al., 2011). These increases may be contributed by a low rate of basal microglia proliferation (Lawson et al., 1992; Ajami et al., 2007) or otherwise a slow incremental recruitment of monocytes or macrophages from the periphery. The significance of increasing microglial numbers is unclear—one hypothesis suggests that it may be a compensatory mechanism to maintain overall function as each individual aged microglial cell declines in function with aging (Streit and Xue, 2010). The increase may also be driven by a cumulative history of environmental influences (e.g., infections, injuries, inflammatory insults) that have triggered episodes of microglial proliferation that did not fully revert back to the basal level (Ajami et al., 2007). Currently, functional significance of increased microglial numbers remains unknown.

With aging, the order and regularity of the mosaic distribution of microglia in the CNS also appears to deteriorate. Microglia in the aged mouse cortex are distributed less evenly than in the young cortex; neighboring cells occasionally have somata that are closely juxtaposed, instead of being evenly spaced and clearly separated (Tremblay et al., 2012). In the healthy young retina, microglia have an unique distribution as horizontally tiled arrays that are limited only to the laminated inner layers of retina, with no microglia found in the outer retinal layers (Santos et al., 2008). With aging, this distribution pattern breaks down as microglia translocate into the formerly “microglial-free” zone of the outer retina and accumulate in the subretinal space (Xu et al., 2008; Damani et al., 2011). The consequences of aging-related changes in microglial distribution are not generally obvious; however in the retina, this redistribution brings microglia into novel contact with photoreceptors and retinal pigment epithelial (RPE) cells, initiating immune changes in those cells that recapitulate pathologic phenotypes in AMD (Ma et al., 2009, 2012). Finding out what drives age-related microglia redistribution may be therapeutically relevant to AMD treatment and prevention (Karlstetter et al., 2010; Buschini et al., 2011). These studies comparing the distribution of microglia in young and aged animals rely on the ability of microglial markers such as Iba1 to consistently reveal microglia across different ages. Although it is theoretically possible that unknown subsets of microglia may change their expression of these markers with aging to elude detection and confound the results described, the stability of Iba1 expression across aging (unpublished data) and different activation states, makes this unlikely.

In addition, microglia undergo changes in their ramified morphology with aging. In the mouse retina, aged microglia demonstrate smaller dendritic arbors relative to young microglia which are less branched and are have smaller total process lengths (Damani et al., 2011). In the aging mouse cortex and hippocampus, microglial dendritic arbors are similarly smaller, more variable in size, less circularly symmetric, and more elongated in shape (Sierra et al., 2007; Tremblay et al., 2012). In aged human (but not in rodent) brains, sporadic microglial cells have been observed to exhibit dystrophic morphologies in which dendritic arbors appear deramified, with residual processes showing increased tortuosity, cytoplasmic beading, and fragmentation, reflective of ongoing cytorrhexis (Streit et al., 2004). As microglial processes are constitutively dynamic structures whose contact with surrounding cells mediate a host of functions including immune surveillance (Nimmerjahn et al., 2005), synaptic regulation (Wake et al., 2009; Tremblay et al., 2010; Schafer et al., 2012), regulation of neuronal activity and neurogenesis (Nakanishi and Wu, 2009; Nakanishi et al., 2011), these age-related reductions in the structure of ramified processes are likely to be functionally influential and indeed detrimental to the efficiency of these functions. A summary of changes in microglial distribution, morphology, and behavior associated with aging is provided in Table 1.

**Table 1:** **Summary of changes in microglial distribution, morphology, and behavior associated with aging**.

**Phenotypes**
**CHANGES IN MICROGLIAL DISTRIBUTION**
Increase in microglial numbers/density in neural parenchyma
Decrease in regularity in distribution
Translocation into areas not previously occupied by microglia (e.g., to the outer layers of the retina)
**CHANGES IN MICROGLIAL MORPHOLOGY**
Decrease in individual microglial ramification (dendritic arbor area, branching, and total process length)
Appearance of morphological changes suggestive of increased activation state (e.g., perinuclear cytoplasmic hypertrophy, retraction of processes)
Sporadic appearance of dystrophic microglia in aged human brains
**CHANGES IN MICROGLIAL DYNAMIC BEHAVIOR**
Decrease in rate of process movement
Decrease in rate of migration to focal tissue injury

### Aging changes in basal states of microglial activation

In addition to changes in anatomy, microglia demonstrates aging changes in their immune physiology. Multiple lines of evidence have indicated that microglia in the aged CNS show increased basal states of activation. In histopathology studies of the aging human brain, microglial morphologies exhibit a perinuclear cytoplasm hypertrophy and retracted processes, reminiscent of activated microglia (Sheng et al., 1998; Miller and Streit, 2007). Aged microglial immunophenotypes also resemble those of activated microglia, with increased expression of major histocompatibility complex II (MHCII) and CD11b (Rogers et al., 1988; Perry et al., 1993; Frank et al., 2006; Ziv et al., 2006). Molecular markers typically found to be up-regulated in activated microglia activation (e.g., Iba1, OX6) are also increased in aged microglia in the absence of injury or disease (Ogura et al., 1994; Frank et al., 2006). In healthy aged humans, *in vivo* positron emission tomography (PET) using R-[^11^C]PK11195 revealed increased ligand binding in several cortical and subcortical areas, indicating increased levels of basal microglial activation (Schuitemaker et al., 2010).

Consistent with these markers of increased activation, aged microglia are found to express increased levels of effector molecules associated with activated microglia. Increased expression of inflammatory cytokines (such as IL1β, TNF-α, IL6) are detected in aged microglia studied *in situ* (Sheng et al., 1998), isolated *ex vivo* (Sierra et al., 2007; Njie et al., 2012), or when cultured *in vitro* (Ye and Johnson, 1999). Interestingly, studies examining the levels of anti-inflammatory cytokines such as IL10 are less uniform, with differing associations with aging described in different studies (Ye and Johnson, 2001; Sierra et al., 2007). On the whole, there is consensus on the increased activated basal state of microglia in healthy aging, indicating that aged microglia may contribute to the graded chronic states of “para-inflammation”(Medzhitov, 2008) that is associated with the increased susceptibility of the aged CNS to neurodegenerative diseases in which chronic neuroinflammation feature.

## Influence of aging on everyday functions of microglia

### Aging microglia and neurotrophic functions

Given the above descriptions for how microglia change in their structural and basal activation state with aging, how can we connect these aging phenotypes to potential changes in “everyday” functions of microglia? Concepts concerning the constitutive functions of microglia in a healthy CNS have been changing and developing in an exciting way over the last few years as key discoveries reveal new functional roles. A number of metaphors have arisen in the literature concerning these roles: microglia, for their role in dynamic immune surveillance, have been likened to “cops on the beat” (Raivich, 2005). For their role in constitutive pruning and maintenance of synapses, they have been named as “constant gardeners” (Hughes, 2012). Microglia also appear to maintain a conducive environment in the CNS for the healthy function and survival of neurons, prompting the description of “industrious housekeepers” (Streit and Xue, 2009). With regards to the latter function, the presence of ramified microglia have been associated with the provision of trophic support for neurons; neuronal cell death in injury models increases with maneuvers that result in the depletion of microglia (Montero et al., 2009; Vinet et al., 2012). Conversely, repletion of microglia or introduction of exogenous microglia result in neuronal rescue in injury models (Kitamura et al., 2004; Imai et al., 2007), driven possibly by microglial production of trophic influences such as growth factors (Imai et al., 2007; Boscia et al., 2009), adenosine (Lauro et al., 2010), TNF-α (Lambertsen et al., 2009), or via the clearance of deleterious byproducts of neurotransmission (e.g., glutamate) (Persson et al., 2005; Persson and Ronnback, 2012) and metabolism (e.g., iron and excess oligodendroglial membrane) (Fitzner et al., 2011; Ward et al., 2011). As such, the senescent debilitation of microglia, suggested by the emergence of dystrophic, fragmented microglia in aged human brains, have been associated with a decline of neuroprotective function, and with it, an increased vulnerability of the CNS. Indeed, the so-called “microglial dysfunction hypothesis” (Streit and Xue, 2009) articulates that the age-related susceptibility to neurodegenerative disease in human is causally connected to the age-related microglial deficiency in neuroprotective function. In support of this view, dystrophic microglia have been co-localized with areas of neurodegeneration and tau pathology in Alzheimer's brains (Streit et al., 2009), indicating that neurodegeneration in these examples follow from the failure of local support from senescent microglia.

### Aging microglia and maintenance of neuronal activity and synapses

As the “constant gardener” of synapses, microglia in recent studies have been found to constitutively regulate synaptic structure and neuronal activity. The constant dynamic behavior of microglial processes (Davalos et al., 2005; Nimmerjahn et al., 2005) appears not to be a random, cell-autonomous feature but are instead have their overall rate, extent, and direction regulated and guided by neuronal activity (Fontainhas et al., 2011; Li et al., 2012). These dynamic processes are observed to make repeated contact with synaptic structures and influence synaptic stability and pruning in an activity-dependent manner (Wake et al., 2009; Tremblay et al., 2010; Paolicelli et al., 2011; Schafer et al., 2012), potentially altering overall activity patterns in neuronal networks (Pascual et al., 2012). Microglia are capable of modulating the activity of neurons by directly contacting neuronal somata with their processes, down-regulating the activity of contacted neurons (Li et al., 2012). Taken together, these forms of regulation seem to constitute a homeostatic mechanism regulating overall levels of activity in the CNS; neurons with greater levels of activity release the attractant ATP in an activity-dependent manner via probenecid-sensitive pannexin-1 channels (Fontainhas et al., 2011; Li et al., 2012), inducing microglia to migrate and polarize their processes toward them, and which are then reduced in their activity following microglial contact.

Is this “gardening” function of microglia influenced by aging? In our work, we have observed that aged microglia interestingly decline in their dynamic motility of their processes and in their migration rates through neural parenchyma (Damani et al., 2011). These decrements may translate into less frequent and extensive synaptic and neuronal contacts and prolonged microglia response times. These may potentially be related to (1) age-related changes in the structure and electrotonic properties of dendrites and dendritic spines (Duan et al., 2003; Kabaso et al., 2009; Morrison and Baxter, 2012) and (2) a decreased homeostatic regulation of neuronal activity. Also, we had found that microglia migration to sites of neural injury is slower to initiate in aged animals—but once accomplished, microglial aggregation tends to be more sustained and less reversible. Dysregulated fluxes of microglia can therefore result in more sustained imbalances in activity homeostasis, and possibly contribute to differential responses to excitotoxic damage observed in young versus aged brains (Campuzano et al., 2008).

### Aging microglia and adult neurogenesis

An additional constitutive role that microglia play involves the regulation of adult neurogenesis. It is known that adult neurogenesis diminishes with aging, driven perhaps by a declining pool of the neural stem cells (Medrano et al., 2009; Encinas and Sierra, 2012), or a cell-autonomous loss of proliferative capacity in precursor cells (Kuhn et al., 1996). However, as microglia are capable of influencing neurogenesis *in vitro* studies (Butovsky et al., 2006; Walton et al., 2006; Cacci et al., 2008) and are associated with altered neurogenesis *in vivo* studies (Ekdahl et al., 2003; Monje et al., 2003), the contribution of aging microglia to this decrease has been examined. In one study in which neurosphere formation from dissociated hippocampus tissue was used as a measure of neuronal precursor cell (NPC) activity, lower levels of neurogenesis was found in 9-month-old mouse hippocampal tissue relative to 2-month-old tissue (Vukovic et al., 2012). While depletion of microglia from hippocampal tissue did not alter neurogenesis at 2 months, it resulted in increased neurogenesis at 9 months, indicating that microglia in the aged hippocampus, but not in the young hippocampus, contribute to an age-related suppression of NPC activity.

What controls the ability of microglia to influence neurogenesis? The ability of aged microglial to limit neurogenesis has been related to the increasing pro-inflammatory basal state of aging microglia, in which pro-inflammatory mediators produced by aged microglia can inhibit neurogenesis (Ekdahl et al., 2003; McPherson et al., 2011). The connection between microglia and neurogenesis is, however, complicated by findings that different subtypes of microglia in the CNS may affect neurogenesis differentially. Depletion of all microglia from dissociated hippocampi of young, exercising mice reduces neurosphere formation *in vitro*, suggesting that the entire population of microglia post-exercise exerts a net positive contribution to neurogenesis (Vukovic et al., 2012). However, depletion of only the Csf1r^+^MHCII^+^ subpopulation of microglia conversely increased neurosphere formation, indicating that this subset may instead hold back neurogenesis. These results present the possibility that subpopulations of pro- and anti-neurogenesis microglia change in their representation as a function of aging and may differentially regulate neurogenesis in different brain areas. Also, microglial effects on neurogenesis may also be modulated by signals in the aging brain environment. CX3CL1-CX3CR1 signaling in particular appears influential in conferring on microglia their pro-neurogenesis effects (Bachstetter et al., 2011). CX3CL1 levels in the aged hippocampus were found to decline in concert with decreasing rates of neurogenesis. Revealingly, exogenous CX3CL1 application was found to increase neurogenesis, and is effective only in the presence of microglia (Bachstetter et al., 2011; Vukovic et al., 2012).

The types of communication occurring between microglia and neural precursor cells (NPC) are not fully understood. Microglia can influence NPCs via secreted factors that direct their migration and differentiation (Aarum et al., 2003; Walton et al., 2006). These may occur either by direct signaling or by modifying the microenvironment of the neurogenic niche (Battista et al., 2006). Alternatively, microglia can physically interact with neural precursors by direct contact, influencing neurogenesis by phagocytosis of apoptotic newborn neurons (Sierra et al., 2010). However, this form of microglial phagocytosis appears unaffected by aging or by microglial activation, further reflecting a nuanced relationship between microglial function and neurogenesis.

## Influence of aging on microglia responses to perturbations

In the above sections, we have largely considered how the aging phenotype in microglia influences their functions in a healthy uninjured CNS. The question that is considered in this section concerns how aging in microglia influences their ability to respond appropriately to perturbations such as injury, infections, or disease. The classic notion concerning microglial response to perturbation is the acquisition of an activated state. Microglia demonstrates a particular activation status that is a function of the balance of extrinsic signals in their milieu (Hanisch and Kettenmann, 2007). These signals are composed of either “On” signals that induce greater activation, or “Off” signals that induces the maintenance of, or a return to, a resting basal state (Van Rossum and Hanisch, 2004; Biber et al., 2007). Also relevant to this picture are intrinsic, cell-autonomous factors and mechanisms within microglia that influence and limit the magnitude and duration of activation responses. While these mechanisms have not been fully characterized, they include nuclear receptor signaling (Saijo et al., 2011, 2013) and microRNA-regulated gene expression (Ponomarev et al., 2013). Optimal microglial responses to perturbation comprise of a rapid, pro-inflammatory activation, necessary to produce efficient responses to injury and infections, as well as a well-timed and expeditious resolution of activation, required to avoid sustained damage to the CNS.

There is accumulating evidence that microglial responses to CNS perturbations become more dysregulated with aging. In the aged brain, in addition to the mild increases in basal microglial activation, microglia demonstrates increased priming or sensitization, and as a result generates responses to inflammatory stimuli that are exaggerated and prolonged. In numerous studies involving animal models of infectious and inflammatory challenge (Sparkman et al., 2005; Sierra et al., 2007; Abraham et al., 2008; Barrientos et al., 2009a,b; Norden and Godbout, 2012), hemorrhagic stroke (Wasserman and Schlichter, 2008; Wasserman et al., 2008), cognitive and physiological stressors (Buchanan et al., 2008; Rosczyk et al., 2008), and neurological injury induced by axotomy (Conde and Streit, 2006), neurotoxins, (Sugama et al., 2003) or trauma (Sandhir et al., 2008), responses generated in aged animals tend in general to be larger and more sustained, and culminate in more severe anatomical degeneration and functional debilitation relative to those produced in young animals. These larger inflammatory responses appear to be causally related to increased neurological deficits; in studies involving *E. coli-* and lipopolysaccharide (LPS)-induced systemic infection in aged mice, inhibition of IL-1β signaling blocked both the neuroinflammatory response as well as the associated cognitive deficits (Frank et al., 2010; Barrientos et al., 2012). Augmented neuroinflammatory responses are thought to suppress growth factor signaling, particularly that involving BDNF (Barrientos et al., 2004; Chapman et al., 2012), which is directly associated to neurological dysfunction.

What mechanisms result in age-related priming of microglia? An altered balance of “On” vs. “Off” extrinsic signals in the aging brain milieu, as well as the altered reception of these signals by microglia, constitute potential mechanisms. Microglial expression of pattern recognition receptors (PRRs) such as TLR1, TLR2, TLR4, TLR5, TLR7, and CD14 are up-regulated with increasing age (Letiembre et al., 2007). “Off” signaling involving CX3CL1-CX3CR1 and CD200-CD200R signaling also undergoes aging changes as the expression levels of “Off” ligands, CX3CL1 (Bachstetter et al., 2011) and CD200 (Frank et al., 2006; Lyons et al., 2007), as well as “Off” receptor CX3CR1 (Wynne et al., 2010), all decrease with advancing age. These reductions in “Off” signaling in aged microglia may be the basis for a more chronic activation and prolonged pro-inflammatory responses, a phenotype that is milder but mechanistically similar to the more pronounced pro-inflammatory effects observed in CX3CR1- and CD200-knockout mouse models (Hoek et al., 2000; Corona et al., 2010). The ability of exogenous CX3CL1 to reverse phenotypes associated with microglial aging (decreased neurogenesis, activated immunophenotype) also underscore its involvement as a driver of age-related priming of microglia (Bachstetter et al., 2011).

ATP signaling via purinergic receptors on microglia surfaces may also contribute to aging-related responses. It has been found that microglial responses to local tissue injury in the forms of increased process dynamism and polarization and directed migration are mediated by the local release of ATP as an injury signal (Davalos et al., 2005; Farber and Kettenmann, 2006; Haynes et al., 2006). In previous work, we found that dynamic microglial responses to the changes in ATP concentrations in the environment are age-related (Damani et al., 2011). As different P2 purinergic receptors have been described to vary with age (Crain et al., 2009; Damani et al., 2011), changes in the reception of ATP-related signals by microglia may contribute to changing dynamic responses in aging microglia.

## What are some drivers of microglial senescence?

What mechanisms result in the development of senescent phenotypes in microglia? A number of theories have been proposed (summarized in Table 2). One theory invokes age-related replicative senescence, which refers to the finite capability of cells to undergo repeated cycles of replication. Microglia may undergo low levels of replication under steady state conditions (Lawson et al., 1992) and can proliferative quickly in response to perturbations (Ajami et al., 2007). In the course of a lifetime, these cycles can culminate in the shortening of telomeres and the attainment of replicative senescence (Olovnikov, 1996). Microglia induced to replicate *in vitro* do in fact demonstrate progressive telomere attrition (Flanary and Streit, 2004), and telomere lengths in microglia are shorter in aged compared to young brains (Flanary et al., 2007). Levels of telomerase, an enzyme that can extend the length of telomeres, also decrease in microglia with age, indicating that aging microglia cannot as successfully re-lengthen telomeres following injury-activated replication (Flanary and Streit, 2005). While replicative senescence in microglia may constrain their ability to go on proliferating, how it can drive the emergence of other senescent microglial phenotypes is unclear.

**Table 2:** **Summary of proposed drivers of microglial senescence**.

**Drivers**	**Effects**
Shortening of telomeres	Induction of replicative senescence and decreased ability to proliferate
Accumulation of intracellular lipofuscin	Decreased autophagy, leading to decreased organelle (e.g., mitochondria) turnover, increased ROS production, increased microglial activation
Accumulation of mtDNA mutations	Dysfunction in respiratory chain and over-production of ROS, leading to increased microglial activation
Increased iron load	Increased intracellular iron leads to increased ROS production and microglial activation

Alternately, microglia aging may result from the accumulation of biological changes that build up within microglia as a consequence of physiological activities sustained over time. Microglia in the aged brain accrue prominent amounts of autofluorescent lipofuscin (Sierra et al., 2007; Tremblay et al., 2012), that appears on ultrastructure studies as lysosomal inclusions (Peinado et al., 1998; Tremblay et al., 2012). Aged retinal microglia located in the subretinal space also demonstrates autofluorescent lipofuscin granules which increase in number with age (Xu et al., 2008). This intracellular accumulation is thought to result from microglia phagocytizing lipofuscin or its precursor molecules from nearby cells; autofluorescent granules found in subretinal microglia have similar spectral characteristics as those found in adjacent RPE cells, suggesting their common origin. Age-related lipofuscin buildup in microglia may indeed be influential on microglial physiology. In recent work, we found that cultured retinal microglia accumulating A2E, a prominent component of ocular lipofuscin, (1) developed a less ramified and more activated morphology, (2) demonstrated changes in gene expression that favored a more pro-inflammatory profile with an increased M1/M2 polarization, and (3) exhibited changes in the expression of complement regulatory proteins that promoted increased complement activation(Ma et al., 2013), all of which correspond to the basal state of increased activation typical in senescence.

Another candidate mechanism invokes the concept of oxidative stress. Microglia are prominent producers of oxidative products including reactive oxygen species (ROS) in the CNS. Mitochondria-derived ROS are produced in close proximity to mitochondria DNA (mtDNA) which encode components of the mitochondria electron transfer complexes. In aged microglia, mtDNA damage accumulates and leads to dysfunction in the respiratory chain and an over-production of ROS (Corral-Debrinski et al., 1992; Lin et al., 2002). Concurrently, decreased microglial autophagy due to age-related accumulation of lipofuscin slows down the turnover of senescent mitochondria, adding to the increase in oxidative stress within microglia (Kurz et al., 2008). As the activation of NF-κB mediated transcription in microglia, which regulates the expression of multiple pro-inflammatory cytokines, is positively modulated by ROS (Toledano and Leonard, 1991), this increased redox state may serve to prime microglia responses to perturbations, resulting in augmented microglial activation states observed in aged microglia (Nakanishi et al., 2011).

The age-related increase of oxidative stress as a driving force for microglia senescence has also been related to iron management in aging microglia. Levels of iron, and its storage protein, ferritin, increase with age in brain tissues (Benkovic and Connor, 1993; Bartzokis et al., 1997; Kwan et al., 2012). Microglia, which express ferritin, are thought to play a role in iron homeostasis (Kaneko et al., 1989; Cheepsunthorn et al., 1998; Widmer and Grune, 2005). As the iron load managed by microglia increases in the aging brain, the risk of oxidative damage to microglia increases as the labile nature of intracellular Fe^2+^ can readily lead to the formation of ROS. Microglial activation with LPS has been shown to increase ferritin expression in microglia and total iron content in brain areas (Hunter et al., 2008). The related natures of increased iron load, ROS production, and increased activation can potentially drive a feed-forward process that accelerates the formation of senescent microglial phenotypes. Indeed, dystrophic microglia in aged brains, as well as those with Alzheimer's and Huntington's disease, have been associated with ferritin immunopositivity and accumulation (Simmons et al., 2007; Lopes et al., 2008), suggesting a link between increased iron load and the development of dystrophy in individual microglial cells.

## Can microglia be rejuvenated?

A discussion of the roles of microglial senescence in brain aging and susceptibility to age-related neurodegeneration leads naturally to the question of whether it is possible to prevent or reverse the aging phenotypes of microglia. Proposed mechanisms for the etiology of microglial senescence and the descriptions of senescent microglial phenotypes present opportunities to formulate therapeutic strategies for achieving this, as well as outcome measures for which these therapies can be assessed. A number of these therapeutic approaches has been discussed or investigated in the literature and are listed thematically in the sections below (Table 3). Although there is currently a dearth of preventative therapies for age-related neurological diseases, research aimed at microglial “rejuvenation” may constitute a fruitful strategy in generating these in the future.

**Table 3:** **Proposed therapeutic approaches for the rejuvenation of senescent microglia**.

**Approaches**	**Effects**
**ANTI-INFLAMMATORY AND ANTIOXIDANT APPROACHES**	
Minocycline	Inhibits microglial activation, decreases microglial proinflammatory cytokine production
IL1RA	Inhibits IL1β-mediated proinflammatory signaling
Dietary supplementation with antioxidants: flavonoids (e.g., luteolin, quercetin, genistein, hesperetin), retinoids/carotenoids (e.g., astaxanthin, crocin, crocetin, retinoic acid, lutein, zeaxanthin), vitamins (E and D3)	Exerts antioxidant and anti-inflammatory effects, decreases markers of neuroinflammation
**PREVENTING OR REVERSING MICROGLIAL “RUN-DOWN” IN AGING**	
Decreasing lipofuscin accumulation with visual cycle modulators (ACU-4429, fenretinide)	Partially inhibits the visual cycle to decrease ocular lipofuscin formation in retinal microglia
Stimulation of microglial autophagy (e.g., anti-lipolytic drugs, rapamycin)	Increases autophagy to promote mitochondria turnover in microglia
Stimulation of TFAM expression or activity (e.g., resveratrol, brimonidine)	Inhibits accumulation of mtDNA mutations in microglial mitochondria, decreasing ROS production
**MODULATION OF THE AGING MICROGLIAL MILIEU**	
Stimulation of CX3CL1-CX3CR1 signaling	Decreases microglial activation states
Stimulation of CD200-CD200R signaling [stimulation of IL4 signaling, fibroblast growth loop (FGL)]	Decreases microglial activation states
Exercise	Decreases microglial activation states, up-regulates proliferation of neural precursor cells
**REPLACEMENT OF AGED MICROGLIA**	
Depletion, followed by autologous or exogenous repletion by bone marrow derived cells	Enables the replacement of endogenous aged microglia with “replacement” immune cells that can carry out microglial functions
Cell-based therapies involving stem cells	Enables the replacement of endogenous aged microglia with “replacement” immune cells that can carry out microglial functions

### Anti-inflammatory and anti-oxidant approaches

The pro-inflammatory and primed characteristics of the aging microglia phenotype prompt the use of an anti-inflammatory approach to alleviate the consequences of microglial aging. Minocycline, a highly bioavailable tetracycline-derived antibiotic with anti-inflammatory properties (Yrjanheikki et al., 1998, 1999; Tikka et al., 2001), has been investigated as an inhibitor of microglial activation in numerous animal models of neuroinflammation and CNS injury (Kim and Suh, 2009). Specific to its effect on aging microglia, minocycline has been shown to ameliorate pro-inflammatory cytokine production and sickness behavior following LPS-administration in aged mice (Henry et al., 2008) and to reduce aging-exacerbated deficits in long-term potentiation (LTP) in the aged rat hippocampus (Griffin et al., 2006; Liu et al., 2012). Clinical studies involving the use of minocycline for neurodegenerative diseases are ongoing; results of completed studies have produced both positive and negative results in separate disease contexts, revealing the need for further investigations (Plane et al., 2010). In contrast to minocycline, which has broad and somewhat varied anti-inflammatory actions, specific and targeted therapeutic approaches have also been considered. The central role of IL1β in mediating age-related pro-inflammatory effects of microglia has led to investigations of the IL1 receptor antagonist, IL1RA, as a potential therapy. Administration of IL1RA was found to ameliorate deficits in long term memory (Frank et al., 2006), impairments in theta-burst late-phase LTP (Chapman et al., 2010), and sickness behavior (Abraham and Johnson, 2009a) following immune challenges by *E. coli* or LPS administration. These findings may prompt future clinical investigations involving approved inhibitors of IL-1 signaling (anakinra, rilonacept, canakinumab) for the treatment of neurodegenerative disease (Dinarello et al., 2012).

As the generation of ROS and increased oxidative stress has been associated with the activation of pro-inflammatory programs in primed microglia (Verri et al., 2012), anti-oxidant approaches involving the use of dietary compounds have been examined in multiple studies, with the goal of reducing microglial activation in the course of reducing oxidative stress (McGahon et al., 1999; Gemma et al., 2002; Abraham and Johnson, 2009b). Flavonoids, a class of plant phenolics found widely in a variety of fruits, vegetables, and grains, have been investigated in the forms of luteolin, quercetin, genistein, hesperetin, among others, and have been found to decrease markers of neuroinflammation (Jang and Johnson, 2010; Izzi et al., 2012). Dietary administration of luteolin, one of the more potent agents in this regard (Comalada et al., 2006), decreased inflammatory markers and improved spatial working memory in aged mice (Jang et al., 2008, 2010). Dietary components of the retinoid/carotenoid class (which include astaxanthin, crocin, crocetin, retinoic acid, lutein, zeaxanthin, and their synthetic derivatives) have also been found to decrease microglia and macrophage activation in numerous *in vitro* studies (Dheen et al., 2005; Xu and Drew, 2006; Kim et al., 2008, 2010; Nam et al., 2010; Bian et al., 2012). Anti-oxidant supplements such as alpha-tocopherol (Vitamin E) (Murray and Lynch, 1998; Berg et al., 2005) and vitamin D3 (Moore et al., 2005) have been found to exert anti-inflammatory benefits. While the combination of *in vitro*, animal model, and epidemiological studies indicates the promise of dietary supplementation, verification of benefit in large scale controlled clinical studies is a necessary next step. Currently, there are few therapies of this nature that have been proven and approved for age-related neurodegenerative disease. One exception is AMD, a disease in which activated microglia in the aged retina feature (Gupta et al., 2003; Ma et al., 2013), for which anti-oxidant supplementation with vitamin A, C, E, and zinc, constitutes the current standard of care in reducing risk for disease progression (Age-Related Eye Disease Study Research, 2001). Results for a large scale trial involving supplementation with the carotenoids, lutein and zeaxanthin, and omega-3 fatty acids, for preventing AMD progression are expected in mid-2013 (AREDS2 Research Group et al., 2012).

### Preventing or reversing microglial “run-down” in aging

The concept that aging microglia accumulate progressive physiological changes (build-up of lipofuscin, increasing number of mtDNA mutations) that drive the emergence of senescent phenotypes suggest the feasibility of strategies that aim to ameliorate or reverse these changes. Measures targeted at lipofuscin accumulation in microglia may help alleviate lysosomal dysfunction, increase autophagy, decrease ROS production, and in general, ameliorate the detrimental phenotypes of aging microglia (Nakanishi and Wu, 2009). In the retina, lipofuscin accumulates in microglia of aged and AMD retinas (Ma et al., 2013) and is compositionally akin to that accumulating in nearby RPE cells (Xu et al., 2008). Current clinical studies of visual cycle modulators (ACU-4429, fenretinide) (Kubota et al., 2012; Mata et al., 2012) that aim to decrease lipofuscin accumulation in RPE cells may also be helpful in decreasing accumulation in retinal microglia. Alternatively, measures that stimulate autophagy, a process slowed by lipofuscin accumulation, may also be helpful. Anti-lipolytic drugs and rapamycin, which are agents found increase autophagy (Donati, 2006; Cai et al., 2012), have also been associated with reductions in age-related oxidative damage (Donati et al., 2006; Yang and Ming, 2012) and microglial activation (Dello Russo et al., 2012), and may therefore be useful in the therapy of neurodegenerative disease (Chong et al., 2012). Early stage clinical trials have been conducted with rapamycin for AMD and multiple sclerosis (NCT00095329 and 00766649) but results are not currently available.

With respect to the age-dependent accumulation of mtDNA mutations in aged microglia, a nucleus-encoded protein called transcription factor A, mitochondrial (TFAM) has been found to promote mtDNA transcription and maintain its architectural structure (Kanki et al., 2004). TFAM overexpression can reduce ROS production and NF-κB activation *in vitro* and decrease age-related mtDNA damage and IL1β expression in hippocampal microglia *in vivo* (Hayashi et al., 2008). As a result, compounds that can stimulate TFAM expression or activity, such as resveratrol (Lagouge et al., 2006; Vetterli et al., 2011) and brimonidine (Lee et al., 2012a) may be useful in this strategy.

### Modulation of the aging microglial milieu

The increased basal inflammatory state and priming in aged microglia that result from an age-dependent shift in balance between “On” and “Off” signals may be another target for microglial “rejuvenation.” During aging, the expression levels of “Off” ligands CX3CL1 and CD200 decrease concurrently with increases in microglial activation status; the causal influence of these changes are demonstrated by the ability of exogenous CX3CL1 or a CD200 fusion protein to ameliorate phenotypes associated with microglial aging (Lyons et al., 2007, 2009a,b; Cox et al., 2012; Vukovic et al., 2012). These results present these signaling pathways as promising molecular targets for intervention. For the CX3CL1-CX3CR1 signaling axis, despite increasing attention in different disease areas, suitable modulatory agents have yet to be investigated in clinical studies (D'Haese et al., 2012). For the CD200-CD200R axis, measures that increase IL4 (phospholipid microparticles-incorporating phosphatidylserine, atorvastatin), which up-regulates CD200 expression, can improve age-related decreases in LTP attributed to microglial aging (Nolan et al., 2005; Lyons et al., 2007, 2009b, 2011; Clarke et al., 2008). Also, a neural cell adhesion molecule (NCAM)-derived peptide, FGL, which can increase CD200 expression *in vitro* and *in vivo*, has been found to abrogate age-related increases in glial activation and synaptic changes (Downer et al., 2009, 2010; Ojo et al., 2012). Discovery of other “Off” signaling pathways can augment this strategy of modulating the milieu of aging microglia in ways that promote microglial quiescence.

### Exercise

Physical exercise has been associated with a number of salubrious effects on CNS in animal studies, including increasing adult neurogenesis, improving performance on cognitive tasks, and ameliorating various declining neurological parameters observed with aging (Van Praag et al., 1999, 2005; Kim et al., 2004; Blackmore et al., 2009). Also, exercise has been implicated in improving aspects of systemic immune function (Woods, 2005). Recent studies have connected these two classes of effects in showing that exercise may exert its effects on the CNS, at least in part, through the modulation of microglia activity. The exaggerated neuroinflammatory response following a peripheral immune challenge in aged animals, which is associated with increased cytokine production, reduced BDNF expression, and increased cognitive deficits, was found to be completely reversed by voluntary exercise (Barrientos et al., 2011). This effect of exercise appears to be mediated by the “de-priming” of aged microglia as microglia from isolated the brains from exercising mice similarly demonstrated a more modest pro-inflammatory response to LPS administered *in vitro* relative to those from non-exercising mice. In a separate study, exercise was found to promote adult neurogenesis by stimulating greater NPC proliferation (Vukovic et al., 2012). These effects again appear to be mediated via microglia as prior depletion of microglia completely eliminates them. In this study, exercise seems to influence microglia through increased expression of CX3CL1, which confers on microglia the ability to up-regulate NPC proliferation. In other studies, exercise in animals was found to decrease age-related basal microglial proliferation (Kohman et al., 2012), and to lower microglial activation in models of hypoxia (Lin et al., 2012), high-fat diet (Yi et al., 2012), tau overexpression (Leem et al., 2011), and closed-head injury (Chen et al., 2012), further supporting the notion that exercise can play a role in deactivating and de-priming microglia. The molecular mechanisms underlying how exercise mediates these effects will be a fascinating area for future study, and may discover agents that can elicit exercise-induced effects without the need for actual exercise.

### Replacement of microglia as therapy?

Although microglia are long-lived residents of the CNS and are not readily turned-over in the uninjured CNS, it remains plausible that aged microglia, instead of being modulated, may actually be wholly replaced or reinforced by exogenously introduced cells capable of carrying out the functions of young, non-senescent microglia. Studies in organotypic hippocampal slice cultures have shown that brain slices deprived of microglia can be again replenished by exogenously introduced cultured microglia which are able to colonize the brain parenchyma, acquire a ramified morphology, and confer neuroprotection to excitotoxic injury (Vinet et al., 2012). Animal studies have demonstrated that lethal irradiation followed by bone marrow transplantation allows the elimination of endogenous microglia and their replacement by bone marrow-derived cells which acquire features of microglia within the CNS parenchyma (Simard and Rivest, 2004). This technique of “microglial replacement” has enabled microglia expressing a mutant version of a particular gene in a transgenic mouse to be replaced by wild type microglia expressing a functional version of the same gene. These microglial “replacements” have been successful in alleviating the neurological and systemic symptoms in *MECP2* mutant mice, a model for Rett syndrome (Derecki et al., 2012), in correcting abnormal grooming behavior in *Hoxb8* mutant mice, a possible model for obsessive-compulsive disorder (Chen et al., 2010), and in increasing survival in *SOD1G93A* transgenic mice, a model for amyotrophic lateral sclerosis (ALS) (Lee et al., 2012b). Conversely, replacement of wild type microglia with transgenic microglia deficient in MyD88 signaling was found to increase disease susceptibility in a mouse ALS model (Kang and Rivest, 2007). Even in the absence of bone-marrow transplantation, the depletion of brain microglia has been found to induce an influx of peripheral monocytes into the CNS that repopulates the microglia-depleted areas (Varvel et al., 2012). These “replacement” cells intriguingly resemble endogenous microglia in distribution, morphology, and physiology, suggesting the notion that worn-out, aged brain microglia can perhaps be replaced by fresher and more able recruits from the periphery. The possibility that we can harness our intrinsic capability to “self-rejuvenate” our CNS microglia using our peripheral immune system is a radical but tantalizing concept.

The ability of microglia to be replenished in the CNS by peripherally derived cells that can take on features and functions of microglia present the possibility that these approaches may be in the future be translated into cell-based therapies to rejuvenating the population of senescent microglia in aged brains. Further studies still remain to be done regarding (1) possible sources of replacement cells (including stem cells sources) (Selvaraj et al., 2012), (2) the physiological functioning of newly integrated cells in an aged brain milieu, and (3) the use of brain conditioning regimens to optimize the spatial and temporal extents of microglial replacement (Capotondo et al., 2012) in order to increase and to realize the feasibility of such strategies.

### Conflict of interest statement

The author declares that the research was conducted in the absence of any commercial or financial relationships that could be construed as a potential conflict of interest.
